# Limiting esophageal temperature in radiofrequency ablation of left atrial tachyarrhythmias results in low incidence of thermal esophageal lesions

**DOI:** 10.1186/1471-2261-10-52

**Published:** 2010-10-26

**Authors:** Armin Sause, Osman Tutdibi, Karsten Pomsel, Wilfried Dinh, Reiner Füth, Mark Lankisch, Thomas Glosemeyer-Allhoff, Jan Janssen, Micheal Müller

**Affiliations:** 1 HELIOS Klinikum Wuppertal, Department of Cardiology, Arrenberger Str. 20, 42117 Wuppertal, Germany; 2 HELIOS Klinikum Wuppertal, Department of Gastroenterology, Heusenerstr. 40, 42283 Wuppertal, Germany; 3 St. Martinus-Hospital, Department of Gastroenterology, Hospitalweg 6, 57462 Olpe, Germany; 4 University Witten/Herdecke gGmbH, Alfred-Herrhausen-Straße 50, 58448 Witten, Germany

## Abstract

**Background:**

Atrio-esophageal fistula formation following radiofrequency ablation of left atrial tachyarrhythmias is a rare but devastating complication. Esophageal injuries are believed to be precursors of fistula formation and reported to occur in up to 47% of patients. This study investigates the incidence of esophageal lesions when real time esophageal temperature monitoring and temperature limitation is used.

**Methods:**

184 consecutive patients underwent open irrigated radiofrequency ablation of left atrial tachyarrhythmias. An esophageal temperature probe consisting of three independent thermocouples was used for temperature monitoring. A temperature limit of 40°C was defined to interrupt energy delivery. All patients underwent esophageal endoscopy the next day.

**Results:**

Endoscopy revealed ulcer formation in 3/184 patients (1.6%). No patient developed atrio-esophageal fistula. Patient and disease characteristics had no influence on ulcer formation. The temperature threshold of 40°C was reached in 157/184 patients. A temperature overshoot after cessation of energy delivery was observed frequently. The mean maximal temperature was 40.8°C. Using a multiple regression analysis creating a box lesion that implies superior- and inferior lines at the posterior wall connecting the right and left encircling was an independent predictor of temperature. Six month follow-up showed an overall success rate of 78% documented as sinus rhythm in seven-day holter ECG.

**Conclusion:**

Limitation of esophageal temperature to 40°C is associated with the lowest incidence of esophageal lesion formation published so far. This approach may contribute to increase the safety profile of radiofrequency ablation in the left atrium.

## Background

Pulmonary vein antrum isolation using radiofrequency ablation has become an effective therapy in symptomatic patients with atrial fibrillation. Additional linear ablation in the left atrium is performed in persistent atrial fibrillation and atrial flutter. Non-lethal complications such as cardiac perforation, stroke and pulmonary vein stenosis have been reported to occur with rates of 1.3%, 0.2% and 1.3% respectively [1,2]. A recently reported complication describes esophageal injury leading to left atrial esophageal fistula. Despite its low incidence (0.03-0.1%) this usually lethal complication is of tremendous clinical importance [3-7]. Other serious esophageal injuries include vagus nerve damage with acute pyloric spasm and gastro-paresis [8]. Different strategies are proposed to avoid esophageal injury. Power and temperature settings are limited when ablating at the posterior wall of the left atrium [9]. Visualization of the esophagus by barium swallow or tagging the course of the esophagus by electro-anatomical mapping has been proposed in order to modify ablation lines in areas of close proximity to the esophagus [10]. A pilot study using an irrigated intraesophageal cooling balloon resulted in a significant reduction of intraluminal esophageal temperature [11,12].

Post ablation esophageal wall changes (erosion or ulceration) are reported to occur in up to 47% of patients [13]. Real time temperature monitoring can detect rapid esophageal heating during radiofrequency ablation [14].

The aim of this prospective study was to investigate the incidence of thermal esophageal lesions when limiting the intraluminal esophageal temperature in radiofrequency ablation of left atrial tachyarrhythmias.

## Methods

### Study population

184 consecutive patients with symptomatic atrial fibrillation or left atrial macro-reentrant tachyarrhythmias scheduled for ablation were included in this study. All procedures were performed after obtaining written informed consent approved by the institutional ethics committee.

### Pulmonary vein isolation

Radiofrequency ablation was performed in conscious sedation (continuous intravenous injection of propofol and intravenous injection of fentanyl) or general anaesthesia (10 patients). All patients underwent a pre-procedural magnetic resonance imaging or a computed tomography scan (13 patients) of the left atrium. A transesophageal echocardiogram was performed within 48 hours prior to the procedure to exclude left atrial thrombi and visualize the intraatrial septum. Using a transfemoral venous approach a multipolar catheter was introduced into the coronary sinus. Transseptal puncture was performed under fluoroscopic guidance using a steerable long sheath (Agilis, St. Jude Medical, Saint Paul, MN, USA), and a Brockenbrough needle. Intravenous unfractionated heparin was administered in boli immediately following transseptal puncture to maintain an activated clotting time of 250-350 s. An anatomic map of the left atrium and the pulmonary veins was created using either the NavX-Ensite system (Endocardial Solutions, St. Paul, USA) in 153 patients or the CARTO XP system (Biosense Webster, Diamond Bar, CA, USA) in 31 patients. The segmented MRI or CT image of the left atrium was fused with the anatomic map using either the Verismo (Endocardial Solutions, Saint Paul, MN, USA) or CARTO Merge (Biosense Websters, Diamond Bar, CA, USA) software.

A 7F esophageal temperature probe (Esotherm, Fiab, Florence, Italy) with 3 consecutive olive shaped thermocouples (distance 10 mm) was inserted orally and advanced into the esophagus. The probe was positioned directly posterior to the left atrium using the NavX-Ensite system or fluoroscopic guidance. The course of the esophagus in relation to the pulmonary venous ostia was described and classified in five groups similar to Kottkamp [10]: Right pulmonary vein (RPV)/RPV ostium/mid posterior wall/left pulmonary vein (LPV) ostium/LPV. In case of an oblique course classification was according to the major course and the area with the least distance to the left atrial wall.

Radiofrequency impulses were delivered circumferentially around each pair of veins approximately 1 cm distal of the ostia using an open irrigated tip ablation catheter (IBI coolpath duo; St. Jude Medical, Saint Paul, MN, USA or Navistar thermocool, Biosense Webster, Diamond Bar, CA, USA). Guided by the temperature probe ablation directly adjacent the esophagus was avoided if possible. Radiofrequency generator settings were as follows: 40 Watt, 48°C, flow rate 30 ml/min. When ablating at the posterior wall of the left atrium power was generally reduced to 30 Watt. The ablation catheter was held in position until local signal reduction occurred. However, the catheter was moved at least every 20 seconds.

**Figure 1:**
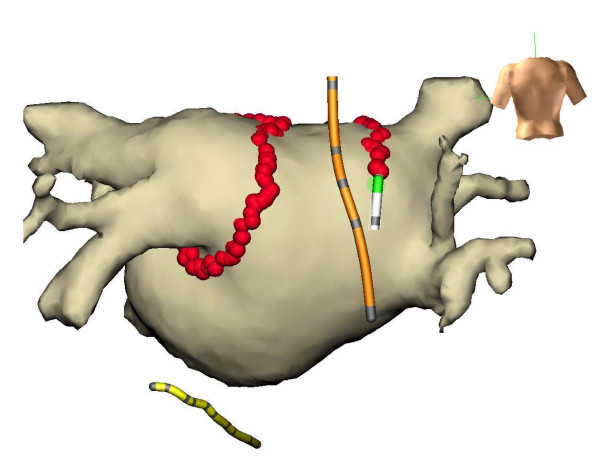
**Ensite NavX procedure with fused MRI of the left atrium**. Temperature probe (orange), ablation catheter (white), coronary sinus catheter (yellow).

During energy delivery the position of the temperature probe was adjusted in a cranio-caudal manner to insure minimal distance of the thermocouples to the tip of the ablation catheter. Figure 1 demonstrates a NavX-Ensite procedure with a fused MRI of the left atrium. The operator continuously monitored esophageal temperature of all 3 thermocouples. When temperature exceeded 40°C ablation was stopped until temperature normalized. Further ablation could be performed using less energy to prevent additional temperature rise. In case of repeated esophageal temperature rise wide encircling or a box lesion (posterior roof- and bottom-line connecting the anterior encircling of the right and left pulmonary veins) could be performed at the operator's choice.

In paroxysmal atrial fibrillation pulmonary vein isolation was considered to be sufficient, whereas in persistent atrial fibrillation a roof-line and a left atrial isthmus-line connecting the left inferior pulmonary vein and the mitral isthmus could be drawn at the operator's preference.

In left atrial macro-reentrant tachycardia entrainment pacing and activation mapping determined the reentrant circuit. In case of previous pulmonary vein isolation procedure the veins were checked for isolation first. If re-connection was documented pulmonary vein re-isolation was performed. Further linear lesions were added to interrupt the macro-reentry when needed.

All veins were checked for isolation with a steerable circumferential decapolar catheter. Bidirectional block evidenced as disappearance of pulmonary vein potentials and exit block when pacing inside the vein with maximum output (10 V/2 ms) confirmed pulmonary vein isolation.

### Esophageal Evaluation

In patients with clinical suspicion of gastro-esophageal reflux disease pre-ablation endoscopy was performed. Ablation was postponed in case of the gastroenterologist's recommendation. No routine endoscopy was performed prior to ablation. All patients underwent endoscopy the day after ablation. Esophageal mucosa at the anterior wall of the mid-esophagus adjacent to the left atrial posterior wall was defined as normal, erythema or ulceration. Any additional follow-up was performed at the gastroenterologist's discretion.

### Follow-Up

Patients were seen in an outpatient clinic for follow-up at three and six months or whenever symptoms occurred. Seven-day Holter ECG was performed at three and six months. Cardiac MRI or trans-esophageal echocardiogram was carried out at six months to exclude pulmonary vein stenosis.

Oral anticoagulation was maintained for at least three months following ablation and according to the CHADS_2 _score thereafter.

### Statistical analysis

All analyses were performed using SPSS statistical software (SPSS 17.0, Chicago, IL). The data are presented as mean ± SD unless otherwise specified. An α = 0.05 was considered statistically significant. Comparison of 2 groups was performed by 1-way analysis of variance (ANOVA). We first analyzed associations without any adjustments and then with adjustments for potential confounders by logistic regression for categorical variables.

## Results

Patient demographics (age, sex), disease characteristics (type of arrhythmia, underlying heart disease, left ventricular ejection fraction, left atrial size, number of ineffective antiarrhythmic drugs and index versus redo ablation procedure) are shown in table 1.

**Table 1:** Patient and disease characteristics

Patient and disease characteristics	
Patients (n)	184
Male (n)	140
Age (years)	58 ± 12
Left atrial parasternal diameter (mm)	42 ± 4.9
Left ventricular ejection fraction (%)	56 ± 16
Paroxysmal atrial fibrillation (n)	129
Persistent atrial fibrillation (n)	43
Left atrial macro-reentrant tachycardia (n)	12
Ineffective antiarrhythmic drugs (n)	
None	27
One	117
Two	30
Three	10
Underlying heart disease (n)	
None	41
Arterial Hypertension	115
Coronary artery disease	21
Dilative cardiomyopathy	13
Index ablation procedure (n)	122

Preablation endoscopy was performed in 23 of 184 patients. Eight patients showed mild reflux disease, three patients hemorrhagic gastritis, and four patients pyloric ulcers. All patients were treated with proton pump inhibitors. Ablation was postponed in all patients with hemorrhagic gastritis and pyloric ulcers until healing was confirmed. Eight patients had no pathologies on pre-ablation endoscopy.

**Figure 2:**
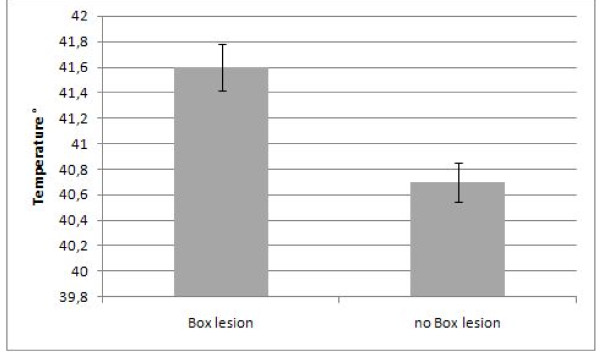
**Mean maximal esophageal temperature (°C) in patients with and without box lesion**. *significant p < 0,05.

The temperature threshold of 40°C was reached in 157 of 184 patients (85%). Despite stopping energy delivery further increase in esophageal temperature was observed. This temperature overshoot was registered in 146 of 157 patients (93%) in whom energy delivery was stopped due to esophageal temperature rise. The mean maximal recorded temperature was 40.8°C in all patients. The maximal recorded temperature was 45.3°C in one patient. Using a multiple linear regression analysis including box lesion, roof-line, mitral-isthmus-line, course of the esophagus, type of arrhythmia and index procedure, box lesion (Beta = 0.29, p = 0.004) remains a significant predictor variable of maximal esophageal temperature. Figure 2 illustrates the mean maximal temperature in patients with and without box lesion. Although the course of the esophagus turned out not being an independent predictor of max temperature, post hoc Bonferroni analysis showed a significantly higher max temperature in the LPV ostia group and the RPV ostia group as compared to the mid posterior left atrium group (table 2). No patient reported about thoracic discomfort such as dysphagia or odynophagia following ablation. Endoscopy showed thermal ulcerations at the anterior wall in the mid esophagus in three patients. This was the index pulmonary vein isolation procedure to treat paroxysmal atrial fibrillation in all three patients. Table 3 summarizes the procedural findings in patients with esophageal ulcerations.

**Table 2:** Esophageal course and maximal esophageal temperature

Esophageal course	LPV	LPV Ostia	Mid posterior left atrium	RPV Ostia	RPV	Total	p value
Total number (%)	19 (10%)	76 (41%)	35 (19%)	49 (27%)	5 (3%)	184	
Mean maximal temperature (°C)	40.0 ± 2.8*	41.2 ± 1.6#	40.1 ± 1.8#,+	41.2 ± 1.4*,+	40.8 ± 1.8*	40.8 ± 1.8	0.005*
Post hoc Test Bonferroni		#p = 0.021		+p = 0.039			

*significant p < 0,05

**Table 3:** Procedural findings in patients with esophageal ulceration

	Esophageal course	Maximal esophageal temperature (°C)	Power reduction (Watts)	Ablation procedure
Patient 1	Mid posterior left atrium	41.1	30	PVI without additional linear ablation
Patient 2	Right pulmonary vein ostium	43.5	20	PVI without additional linear ablation
Patient 3	Right pulmonary vein ostium	42.0	30	PVI without additional linear ablation

PVI: Pulmonary Vein Isolation

**Figure 3:**
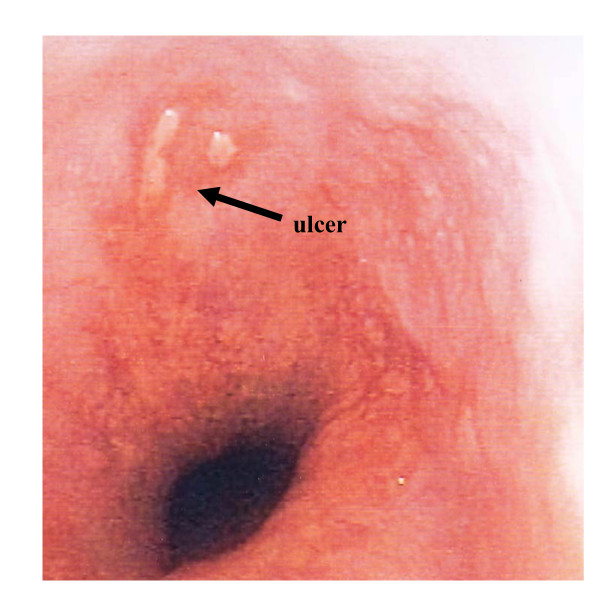
**Endoscopic view of the esophageal ulceration in patient 1**.

An endoscopic picture of the esophageal ulceration in patient one is shown in figure 3. Lesion regression was documented after four to six days and complete lesion healing confirmed after two weeks in all three patients. No patient developed clinical signs of atrio-esophageal fistula on follow-up. Patient, disease and procedural characteristics had no influence on the incidence of esophageal ulcer formation (table 4).

**Table 4:** Ulcer formation and patient, disease and procedural characteristics

	Ulcer	No ulcer	p
**Number of patients (%)**	**3 (1.6%)**	**181 (98.4%)**	

Male (n)	2	138	0,56*
Age (years)	59 ± 1.4	58.1 ± 12	0,54*
Paroxysmal atrial fibrillation (n)	3	127	0,52*
Persistent atrial fibrillation (n)	0	43	
Macro-reentrant tachycardia (n)	0	12	
Index procedure (n)	3	120	0,29*
Redo procedure (n)	0	81	
Mean maximal esophageal temperature (°C)	41.6 ± 1.5	40.8 ± 1.8	0.42*
Ostial course of the esophagus (n)	2	123	0.69*
Non ostial course of the esophagus (n)	1	58	

* not significant

### Complication and follow-up

There were two pericardial tamponades that could be successfully drained with pericardiocentesis. One patient experienced transitory ischemic attack five days after the procedure. One patient showed an asymptomatic 50% stenosis of the left inferior pulmonary vein at six months follow-up.

A blanking period of six weeks after ablation was defined. Arrhythmia recurrence in this period was not considered failure of the procedure. The overall success rate was 78% (145/184 patients). Seven-day holter ECG at six months follow-up confirmed sinus rhythm in 125 of 184 patients off anti-arrhythmic drugs. 20 patients showed sinus rhythm with continued and previously ineffective anti-arrhythmic medication.

## Discussion

Atrio-esophageal fistula is a rare but devastating complication of radiofrequency ablation in the left atrium. Thermal esophageal lesions are believed to be precursors of fistula formation. Little is known about the incidence of thermal esophageal lesions. In most studies less than 100 patients are included and different ablation protocols are used [13,15,16].

Recently Halm reported of 185 patients undergoing left atrial ablation using an open irrigated tip ablation catheter with 30 Watts at the posterior wall. Esophageal temperature monitoring without temperature limitation was utilized. Post-procedural endoscopy of the esophagus revealed ulcer like thermal lesions in 14.6% of patients [17]. Our series of 184 consecutive patients showed an incidence of 1.6% thermal esophageal injury. This is the lowest rate of esophageal lesion formation described so far. No progression to atrio-esophageal fistula occurred. An intraesophageal temperature limit of 40°C appears to be an effective means of reducing the incidence of thermal esophageal damage. Halm did not use a temperature limit and consequently the measured mean maximal temperature was higher than in our series (42.6 ± 1.7°C vs 41.6 ± 1.5°C in patients with ulcers and 41.4 ± 1.7 vs 40.8 ± 1.8°C in patients without ulcers). In accordance to their findings we did not detect ulcers on endoscopy when the maximal temperature stayed below 41°C.

We decided to use the esotherm temperature probe, because it consists of three independent thermocouples with rapid temperature detection capacities. Singh used a different temperature probe with a single thermocouple in 67 patients. When limiting the temperature to 38.5°C they observed 6% ulcerations as compared to 36% without temperature monitoring [15]. It is of great importance to ensure a minimal distance between the temperature probe and the ablation catheter. Using a probe with three thermocouples significantly reduces the chance of missing a temperature rise. In addition the NavX-Ensite system continuously displays the position of the thermocouples in relation to the ablation catheter without the need of fluoroscopy. Ablation directly adjacent to the presumed course of the esophagus was avoided if possible in our study. In spite of applying a lower esophageal temperature limit the higher incidence of ulcerations in the series by Singh could be explained by these differences. However, Singh also used a different ablation protocol with 35 Watts at the posterior wall of the left atrium. This also increases the likelihood of esophageal lesion formation.

Martinek found a total of 2.9% esophageal lesions in 175 patients. Using a maximal power of 25 Watt without esophageal temperature monitoring, additional ablation lines especially an inferior ablation line parallel to the coronary sinus were variables favouring the incidence of esophageal lesions [18]. We could not find a significant correlation of ulcer formation with additional lines or other variables.

The tissue separating the posterior left atrium from the anterior esophageal wall is less than 5 millimetres at its thinnest location [19]. A rapid heat transfer is described [14]. Even though we applied a temperature limit of 40°C a temperature overshoot was observed frequently. The mean maximal esophageal temperature was higher in patients with ulcer formation than in patients without ulcers (41.6°C ± 1.5°C versus 40.8°C ± 1.8°C). However, this difference was not significant, possibly due to the low incidence of lesions. Using a multiple linear regression analysis including roof line, box lesion, mitral isthmus line, type of arrhythmia, redo procedure and course of the esophagus, creating a box-lesion predicted a significant higher esophageal temperature. The box lesion implies an inferior-posterior line connecting the anterior encircling of the right and left pulmonary veins. In this area the esophagus usually shows the closest distance to the left atrial posterior wall.

As stated before the course of the esophagus along the left atrial posterior wall is not an independent predictor of max temperature. However, post hoc Bonferroni analysis revealed a significant higher temperature of 41.2°C in patients with close relationship to either the right or left pulmonary venous ostia as compared to 40.1°C when the esophagus took a course along the mid posterior left atrium.

Although the temperature limit could not be adhered to in most cases due to rapid heat transfer it might be speculated that without this limit even more energy had been applied resulting in more or greater esophageal lesions and eventually an increasing risk of atrio-esophageal fistula formation.

### Limitations

Since endoscopy was not routinely performed prior to ablation it can only be assumed that the described ulcerations in the mid esophagus at the anterior wall are caused by thermal damage. However the typical location far distant from the gastro-esophageal junction makes other causes unlikely.

The diameter of the esophagus by far exceeds the diameter of the temperature probe. An eccentric placement of the probe could lead to an underestimation of esophageal temperature [20]. Furthermore esophageal luminal temperature underestimates esophageal tissue temperature [21]. Using any luminal temperature limit can thus not prevent tissue overheating. Since the temperature limit was not used to up-titrate power but rather to reduce time and energy delivery there was no danger to inadvertently increase energy delivery.

## Conclusions

Limitation of esophageal temperature in radiofrequency ablation of left atrial tachyarrhythmias is associated with the lowest incidence of esophageal lesion formation described until today. Temperature overshoot after cessation of energy delivery is frequent. A posterior box lesion significantly increases the maximal temperatures in the esophagus. Follow-up visits including 7 day holter recordings showed no evidence of reduced efficacy.

Adding temperature limitation in the esophagus to a standard protocol when ablating at the posterior wall of the left atrium may contribute to increase the safety profile of the procedure.

## Abbreviations

MRI: Magnetic Resonance Imaging; CT: Computer Tomography; RPV: Right Pulmonary Vein; LPV: Left Pulmonary Vein; PVI: Pulmonary Vein Isolation

## Competing interests

The authors declare that they have no competing interests.

## Authors' contributions

AS: conceived the study, participated in performing the ablation procedure, and drafted the manuscript. OT: participated in performing the ablation procedure. KP: participated in performing the ablation procedure. WD: performed statistical analysis and participated in the design of the study. RF: participated in the design of the study. ML: participated in the design of the study and corrected the manuscript. TG: carried out the esophageal endoscopy. JJ: carried out the esophageal endoscopy. MM: participated in the design of the study and participated in performing the ablation procedure. All authors read and approved the final manuscript.

## Pre-publication history

The pre-publication history for this paper can be accessed here:

http://www.biomedcentral.com/1471-2261/10/52/prepub
